# From concepts to treatment: a dialog between a preclinical researcher and a clinician in addiction medicine

**DOI:** 10.1038/s41398-022-02177-5

**Published:** 2022-09-21

**Authors:** Youna Vandaele, Jean-Bernard Daeppen

**Affiliations:** Addiction Medicine, Department of Psychiatry, Lausanne University Hospital, Lausanne, Switzerland

**Keywords:** Neuroscience, Addiction

## Abstract

The debate surrounding the brain disease model and the associated questioning of the relevance of animal models is polarizing the field of addiction, and tends to widen the gap between preclinical research and addiction medicine. Here, we aimed at bridging this gap by establishing a dialog between a preclinical researcher and a clinician in addiction medicine. Our objective was to evaluate animal models and the neuroscientific conceptualization of addiction in light of alcohol or drug dependence and treatment in patients struggling with an addiction. We sought to determine how preclinical research influenced addiction medicine over past decades, and reciprocally, what can preclinical researchers learn from addiction medicine that could lead to more effective approaches. In this dialog, we talk about the co-evolution of addiction concepts and treatments from neuroscientific and medical perspectives. This dialog illustrates the reciprocal influences and mutual enrichment between the two disciplines and reveals that, although preclinical research might not produce new pharmacotherapies, it does shape the theoretical conceptualization of addiction and could thereby contribute to the implementation of therapeutic approaches.

## Introduction

YV: The relevance of animal models of alcohol, tobacco, and substance use disorder (referred to as addiction hereafter) is being increasingly questioned [1]. Notably, there is a growing awareness of a translational crisis, evidenced by the poor predictive validity of animal models of addiction [2]. Despite decades of research and considerable progress in our understanding of the neurobiological processes mediating the transition to addiction, most of the promising pharmacotherapies developed in animals failed to prove effective in the treatment of addiction in humans [1]. Besides these unsuccessful efforts, sometimes characterized as a waste of time and resources, preclinical research significantly contributed to the predominance of the Brain Disease Model of Addiction (BDMA) [3], increasingly criticized in the scientific community [1, 4, 5]. In fact, it has even been suggested that viewing addiction as a brain disease could hinder recovery from addiction and promote social injustice [6–8].

However, the influence of the BDMA worldwide, dominating thinking and practice and driven by the neuroscience of addiction, highlights the importance of preclinical research in affecting societal attitudes toward addiction. Preclinical research may not have led to major breakthrough in the development of new pharmacotherapies, but it has generated neurobiological theories that have significantly shaped our conception of addiction with many clinical and societal implications (for better or for worse). My goal here is not to advocate for or against the BDMA, a topic already extensively covered in the literature [1, 4, 9–11]. Instead, I am specifically interested in determining how preclinical research has influenced addiction medicine over past decades and reciprocally, what can preclinical researchers learn from addiction medicine to help develop more effective therapies. To address these questions and to evaluate my models and conceptualization of addiction in light of drug use and treatment in patients struggling with an addiction, I contacted Dr. Jean-Bernard Daeppen, head of the addiction medicine unit at Lausanne University Hospital. We exchanged ideas about the reciprocal influences between preclinical research, neuroscience and addiction medicine and the co-evolution of addiction conceptualization and treatment. We wanted to share this dialog in the hope of establishing more fruitful translational research in the field of addiction to optimize beneficial therapeutic outcomes.

### From theoretical concepts of addiction to the implementation of therapeutic strategies

YV: The current neuroscientific conceptualization of addiction is the result of several decades of preclinical research on the brain mechanisms underlying loss of control over drug (for ease of reading, the term “drug” includes alcohol as well as any pharmaceutical psychoactive substances subject to abuse.) use in animal models of addiction. Much of the progress in preclinical research on addiction emerged in the ’60s from the development of intravenous drug self-administration techniques in monkeys [12] and rats [13]. It was rapidly demonstrated that all drugs abused in humans could be self-administered in non-human animals [14, 15]. Animals could even self-administer the drug to the point of death when offered unlimited access to cocaine [16, 17]. However, researchers rapidly realized that under controlled conditions in which drug intoxication does not interfere with other natural behaviors (i.e., eating and drinking) [18, 19], rats regulate their drug intake and titrate their consumption to reach the desired level of intoxication [20, 21]. Thus, it became evident that drug self-administration was not sufficient to model addiction, and additional tests were required to assess loss of control over drug intake. Preclinical models of addiction were developed to evaluate “addiction-like” behaviors, based on diagnostic criteria of the Diagnostic and Statistical Manual of Mental Disorders (DSM) [22–24] (Box 1).

Building on these multiple models, different theories posit that transition to addiction results from a disruption of brain reward circuits (Box 2) with: an overvaluation of the drug reward relative to alternative nondrug rewards, accompanied by a shift from positive to negative reinforcement (i.e., opponent-process theory) [25–27]; aberrant habitual learning [28, 29]; and heightened sensitivity to drug-associated cues (i.e., incentive sensitization) [30, 31]. On the other hand, impairments in top–down executive control resulting from disruption in the prefrontal cortex can contribute to a loss of goal representation or to deficits in inhibitory control over problematic drug use [32–34]. One common factor between these theories is the central place of compulsion, defined as an irresistible desire or a “force” driving persistent drug use despite negative consequences [35, 36]. This “force”, outside of voluntary control, is sometimes hypothesized to result from the abnormal persistence, dominance and expression of maladaptive habits [28, 29]. In this framework, drug use is automatically triggered in familiar situations or in response to drug-associated cues, without any deliberation, conscious expectation of drug effects or anticipation of negative consequences (i.e., absent goal-directed control). In addition, clinical and preclinical research converge to demonstrate functional disruptions in the prefrontal cortex associated with reduced inhibitory control and broad impairment in executive functions [32, 36], leading some researchers to describe compulsive drug use as resulting from a “defect of the will” [33, 37].

BOX 1 Animal models of addiction **Animal models of addiction**: Preclinical research employs models consisting in mimicking key symptoms of addiction in animals based on diagnostic criteria of the DSM. Most models employ self-administration paradigms in which animals press on a lever or poke their nose into an aperture to earn the drug reward before screening in different tests.

- *The 3-criteria model* consists in measuring (1) the motivation for the drug, (2) the persistence in drug-seeking when the drug is not available and (3) the resistance to punishment [22]. Animals meeting a criterion are the ones ranking in the top 33th centile for this criterion during screening. Animals meeting all 3 criteria are considered as vulnerable to addiction while animals meeting 2 criteria or less are labeled as resilient.
- *The extinction-reinstatement model* is more specifically designed to assess relapse [83]. Following a period of extinction consisting in removing access to the drug, drug-seeking is reinstated by exposing animals to drug-associated cues (cue-induced reinstatement), drug-associated context (renewal), a priming dose of drug (drug-induced reinstatement) or stress (stress-induced reinstatement).
- *The extended access and intermittent access models* manipulate pattern of drug self-administration to reproduce an escalation of consumption [84, 85]. In the first model, the duration of self-administration sessions is increased to 6-h whereas in the intermittent access model, 5 min periods of drug access alternate with 25 min of drug unavailability across 4–12 iterations. Both schedules result in an escalation of consumption and a resistance to punishment with interesting differences; for instance, the intermittent access model produces stronger increase in motivation for the drug and specific pattern of drug-induced dopamine release.
- *Choice models* assess the preference for the drug at the expense of alternative activities [86]. In these models, animals are given a choice between a drug and a nondrug reward. The majority of rats rapidly develop a strong preference for the nondrug reward; However, a minority of individuals maintain a preference for the drug (in general, about 10%) and are considered as vulnerable to addiction.

**Validation of animal models**: Animal models are validated based on three criteria; (1) face validity, (2) predictive validity and (3) construct validity. Face validity is defined as the similarity of what is observed in the animal model vs. what is observed in the human disease. Predictive validity is mainly defined as the ability of the model to correctly predict treatment effects on the human disease. Construct validity refers to the similarity between the mechanisms used in the model to induce de disease phenotype and the disease etiology in human.

**Reverse translation:** The concept of reverse translation was recently proposed by Venniro et al. [2] and consists in using data from humans (i.e., the efficiency of a particular treatment) to develop new animal models that aim at uncovering underlying mechanisms and identifying new treatments.

BOX 2 Neurobiological theories of addiction Neurobiological theories of addiction: Multiple theories of addiction have been proposed over the past decades to describe brain alterations accompanying the development of addiction. Among them, the most influential theories include:

- *The opponent-process theory* [87]—This theory posits that development of addiction would be associated with a transition from positive reinforcement (drug-seeking mediated by the acute positive effects of the drug) to negative reinforcement; the drug is consumed to alleviate the established negative emotional state. In this framework, addiction results from a cycle of spiraling dysregulation of brain reward circuits. With repeated drug use, sensitization and counteradaptation processes in the mesolimbic dopamine, the opioid and the brain and hormonal stress systems contribute to hedonic homeostatic dysregulation, referred to as allostasis, responsible for compulsive use, loss of control over drug-taking and spiraling distress that maintains persistent vulnerability to relapse.
- *The incentive-sensitization theory* [31]—In this theory, the enhancement of mesolimbic dopamine transmission induced by drugs of abuse results in the attribution of excessive ‘incentive salience’ to drug and drug-associated stimuli. Incentive salience is a process that render stimuli attractive by imbuing them with salience. Sensitization of incentive salience results in a transition from drug ‘liking’ to drug ‘wanting’ and is hypothesized to transform ordinary desires for drug experiences into drug craving. This process also contributes to relapse, even after protracted periods of abstinence.
- *The aberrant learning theory* [88]—this theory is based on the role of dopamine as a reward prediction error signal. Dopamine signals an error between the prediction of a reward and the actual reward received. It is argued that, when consumed, drugs mimic the dopamine reward prediction error by increasing dopamine transmission. Thus, according to this theory, repeated dopamine signal across extended drug use would continue to reinforce drug-related stimuli and actions to pathological levels, leading to an overvaluation of drug-seeking and a bias toward drug choice at the expense of alternative rewards.
- *The habit theory* [28]—This theory posits that addiction would emerge from the progressive development and dominance of drug habits over goal-directed control. In this framework, drug use becomes habitual through repeated consumption and association with environmental context and stimuli. With the transition to compulsive drug use and addiction, habitual drug-seeking becomes maladaptive and persists when the drug is no longer pleasurable or when it leads to negative consequences. The persistence of compulsive and maladaptive habit would be explained by drug-induced formation of abnormal habit, mediated by alterations in corticostriatal circuits, and impairment in executive functions (i.e., inhibitory control), mediated by functional disruptions in the prefrontal cortex.

### Jean-Bernard, what is your view, as a clinician, on the influence of this compulsive account of addiction on treatment?

JBD: In the ’60s and ’70s, the clinical approach to addictions was dominated by Alcoholics Anonymous (AA) and Narcotics Anonymous (NA), who consider loss of control and automaticity as landmarks of addiction. AA and NA are built on the belief that individuals are powerless over alcohol and drugs and need the help of a Higher Power to restore control over consumption [38, 39].The AA/NA model and the brain disease model appeared in the scientific literature when the social and political views on addictions were dominated by the moral view that addiction results from the desire for the hedonic effect of alcohol or drugs and from a lack of will to control this desire [40–43]. We can hypothesize that the compulsive account of addiction proposed by neuroscientists was influenced by the AA/NA model of addiction. This model assumes a complete loss of control over drug use and a lack of responsibility in individuals with addictions, and the compulsive account of addiction depicted in the BDMA justifies this claim by providing neurobiological evidence supporting the AA/NA model.

If addiction is conceptualized as a brain disease, characterized by compulsive drug use and a defect of free-will, the logical consequence is that individuals do not have the ability or the responsibility to change. It suggests that external pressures should impose change. In the ’70s, there was a very strong belief that the remission of an addiction was possible only through abstinence, even when the affected individual did not agree [44]. Treatments proposed for alcohol dependence were long hospital stays and disulfiram, while opiate addiction treatment did not consider alternative solutions to withdrawal; both of these reflected some sort of psychological, chemical or physical control imposed on individuals. These confrontational approaches were actually justified by the brain disease concept originating from preclinical research.

Clinical research with experimental treatments testing confrontational counseling styles, including various types of constraint and pressure, became very popular. However, despite this popularity, the efficacy of these approaches has not been conclusive [42, 44]. A systematic review identified 12 studies published between 1972 and 2000 evaluating the efficacy of confrontational counseling for alcoholism treatment; all of them reported no demonstrated benefits [42].

In summary, my impression is that the compulsive account of addiction described in the BDMA appeared to justify that individuals had no responsibility for the occurrence and maintenance of their addictions. However, evidence in my daily practice suggests otherwise. In fact, my patients do not behave like automatons compelled to consume alcohol or drugs, but can instead exert some degree of control over their consumption.

### Youna, is there any evidence of a total loss of control over drug use in animal models of addiction?

YV: There is currently no satisfying evidence of a complete loss of control over drug use in preclinical research. The illusion of compulsion, as defined by an absence of free-will [35], may have arisen from an important limitation in most animal models of addiction [45–47]. In standard ethanol or drug self-administration settings, animals have no other choice but to use the substances available. In these conditions, is drug use symptomatic of a pathological compulsive state or merely an expectable response to lack of choice [47]? The landmark ‘Rat Park’ study of Bruce Alexander and colleagues, have shown that replacing small cages with large naturalistic parks where animals have access to food, play and sex resulted in the preference of plain water over morphine-laced water [48, 49]. Although this experiment comprises some biases, numerous studies have replicated these findings since then and have demonstrated in controlled conditions that providing alternative nondrug rewards during drug self-administration is sufficient to reduce (or even suppress) drug self-administration [50–54]. This was shown with a wide range of drugs (cocaine, methamphetamine, nicotine, alcohol, and heroin) and nondrug rewards (sweet water, food pellets, social interaction, and plain water) under a large array of experimental conditions [50–58].

These findings suggest that drug-seeking can be considered as a voluntary goal-directed behavior, sensitive to changes in environmental contingencies such as the dose, the price, the delay or the availability of alternative rewards. Thus, theories of addiction viewing maladaptive habits and compulsions as central concepts in the etiology of this disorder are now being questioned in both clinical and preclinical literature [59, 60]. Instead, addiction is now increasingly conceptualized as a disorder of choice [61, 62].

Preclinical findings from choice experiments reveal the fundamental role of environmental contingencies on drug use and contribute to the critic of the brain disease model of addiction, which focus on drug-induced brain alterations while minimizing socio-economic and environmental factors.

### Jean-Bernard, is there clinical evidence that echoes these findings from preclinical research?

JBD: Transposed to humans, the “Rat Park” and choice experiments suggest that there is a strong influence of external factors in the induction and maintenance of addictions, and that drug use in addicted patient may continue as a result of a lack of rewarding alternatives. These findings also suggest that remission of addiction in humans is possible when the environment offers interesting alternatives to drug use. There is a large body of research on humans suggesting that the motivation to stop using alcohol or drugs results from the anticipated benefits. For example, in contingency management experiments (Box 3), subjects receive a financial incentive to stop smoking or reduce alcohol and drug use with positive and robust results across numerous studies [63–65]. These studies suggest that patients can exert some degree of control over drug or alcohol use, if circumstances and environmental conditions offer worthwhile alternatives. More generally, preclinical findings from choice experiments suggest clinical research to focus on environmental enrichment (i.e., leisure activities, hobbies, sport, professional activity, …) to promote recovery from addiction. Thus, whether or not addiction is considered as a brain disease, the central role of the environment should be taken into account at every level, from preclinical and clinical research to clinical interventions.

In my experience, contingency management strategies are rarely used to treat addiction. Besides the practical difficulty of implementing them in outpatient settings, part of the problem resides in the fact that, although addiction can be conceived as a disorder of choice, the choice to use or to abstain from using drugs is hard. In my opinion, patients with addictions are prone to strong ambivalence, which is a cardinal feature of addiction.

Ambivalence is characterized by vacillation between the desire to use alcohol or drugs, on the one hand, and regrets when suffering the adverse consequences, on the other hand. In contrast to animals, humans with addictions can verbalize to some degree the feelings and thoughts they experience at different moments in the history of their addiction. In the beginning, subjects do not fight against their progressively increasing consumption. They tend to justify it cognitively (e.g., “I drink like the others, like everyone else, I love wine, it is part of the social life, etc.”). During this period, consumption is part of a routine, the adverse effects are subtle and the motivation to use alcohol or drugs dominates. As the addiction progresses, the desire for drugs becomes stronger in parallel with the increasing costs experienced (i.e., the social, professional, legal and health issues). Therefore, an internal conflict emerges from the opposition between the desire to use and accompanying negative consequences, which finally results in an ambivalence between using or stopping, as expressed by a patient:

« I like to drink. When I don’t have my drink, I miss it and it’s all I can think about. But sometimes I think I should stop. I can’t stand feeling like this, it’s like I have no control. But drinking helps me to relax, it makes it easier for me to talk to people. I don’t know what to do, I feel stuck. I want to, but it’s driving me crazy»

In the example above, the patient manifests this ambivalence, with immediate advantages of using and not getting into treatment, opposed to the delayed costs of doing it. The ambivalence of using fluctuates rapidly during the day, depending on the level of intoxication or symptoms of withdrawal. Typically, the decision to stop and get into treatment is associated with symptoms of withdrawal, while the decision to use typically follows craving. Therefore, the ambivalence could be illustrated by sinusoidal waves with varying amplitudes and periods. In the above case, we observe that the period of the sinusoidal wave is very short, since the patient expresses in the same sentence that he both wants and does not want to use or to stop using alcohol. Therefore, although addiction can be conceived as a disorder of choice where the drug is preferred over alternative activities, it is probably more precise to conceive of addiction as arising from conflicting decisions resulting in an internal fight, and experienced as feelings of ambivalence.

BOX 3 Addiction treatments Available treatments for addiction can be subdivided into three categories:

- Pharmacotherapies—Most effective pharmacotherapies consist in reducing craving and include naltrexin and nalmefen for alcohol dependence or varenicline for nicotine dependence. Other pharmacological tools include agonist treatments for opiate and nicotine dependence, aimed at reducing withdrawal symptoms. Notably, methadone or buprenorphine are used for opiate dependance. For tobacco use disorder, nicotine replacement therapy (patch, gums) can be used to alleviate withdrawal symptoms.
- Psychotherapies—Most popular and well documented evidence-based efficacy treatment include contingency management, cognitive and behavioral therapies and motivational interviewing. Contingency management consists in ‘reinforcing’, or rewarding individuals with voucher, for evidence of positive behavioral changes (i.e., negative urine samples) to reinforce abstinence. Cognitive and behavioral therapy consists in teaching behavioral strategies to help patients (1) learning to recognize and evaluate one’s distortions in thinking, (2) gaining a better understanding of one’s own behavior and motivation, (3) developing problem-solving skills to cope with difficult situations or (4) learning to develop a greater sense of confidence is one’s own abilities. Finally, motivational interviewing is a counseling method that consists in enhancing patients’ motivation to change. The therapeutic hypothesis is that patients need to hear themself talking about changing behavior (change talk) and confront their own behavior to personal values to have enough motivation to change. In contrast to cognitive and behavioral therapy, the therapist doesn’t teach patient behavioral strategies to avoid craving and relapses but guides the patient in his/her elaboration of reasons to change.
- Social support—For successful treatment of addiction, pharmacotherapies and psychotherapies are often combined with different forms of social support including group therapy, community-based organization, alcoholics anonymous or narcotic anonymous, or support from friends and family.

### Youna, do you think that we can explain the neural bases of ambivalence as an opposition between disruption in the brain reward system on the one hand, and alterations in the prefrontal cortex resulting in deficits in top–down executive control, on the other hand?

YV: In fact, the opposition between brain reward circuits and the prefrontal executive system has been suggested to underlie compulsive drug use in many neurobiological theories of addiction [33, 34, 37]. Thus, in my opinion, this general framework cannot explain the internal conflict and ambivalent feelings of your patients. In this framework, repeated exposure to addictive substances alters dopamine signaling in mesolimbic and mesocortical circuits, which results in aberrant learning and an overvaluation of the drug at the expense of alternative nondrug rewards. In parallel, alterations in the prefrontal cortex result in impairments of top–down executive functions and reduce inhibitory control over drug use [34, 36, 66] (Box 2). Thus, alterations at both cortical and subcortical levels contribute to compulsive drug use at the expense of alternative activities and despite negative consequences, leaving no place to explain ambivalence over drug use.

**Fig. 1:**
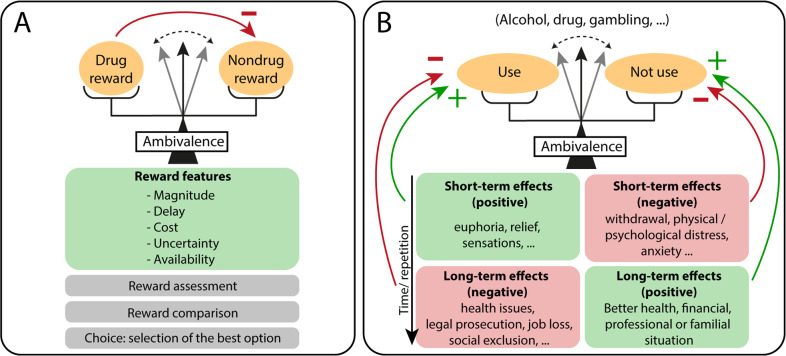
Cognitive processes underlying ambivalence. **A** Ambivalence in a choice between drug and nondrug rewards. In this model, individuals assess the subjective value of each reward based on different features such as reward magnitude, delay, cost, uncertainty, or availability. Then, individuals compare rewards and select the best option. Ambivalence emerges when the two options have comparable value and individuals hesitate between the two. However, it is noteworthy that during addiction, repeated choice of the drug typically interferes with nondrug rewards, resulting in a preference for the drug. **B** Ambivalence in a choice between using and not using the drug. In this model, ambivalence emerges from the double dissociation between the delay (short-term vs. long-term effects) and the valence (positive vs. negative effects) of choice outcome. Individuals could choose to not use the drug when considering the long-term negative consequences of drug use and the long-term benefits of abstinence, but eventually reverse their decision and choose to use the drug, in anticipation of the short-term positive effects (for instance, relief of psychological distress).

Ambivalence refers to the simultaneous existence of contradictory feelings and attitudes, where individuals feel torn between two alternatives (drug vs. nondrug reward or using vs. not using). In the choice model presented in Fig. 1A, the ambivalence results from competing motivations for the drug and a nondrug reward, assessed based on various features such as reward magnitude, delay, cost or uncertainty. However, since repeated drug use tends to interfere with alternative activities (i.e., job loss, divorce, social exclusion …), this would contribute to tilt the balance toward drug use [67], leaving no place for hesitation between the two alternatives. Instead, ambivalence could be better explained by the choice model presented in Fig. 1B. Here, recurrent choices are made between using and not using the drug. Short-term effects of using vs. not using favor drug use, and dysregulation of brain reward circuits occurring during addiction further increases desire for the drug, thereby promoting drug use. However, long-term effects have opposite valence, with negative consequences associated with repeated use while repeated choice of abstinence is benefic. In this model, ambivalence emerges from this double dissociation between delay (short-term vs. long-term effects) and valence (positive and negative effects) of choice outcome. Individuals could choose not using the drug when considering the long-term benefits of abstinence, but eventually decide to use when facing the drug and considering the immediate outcome of this choice.

This double dissociation between delay and valence is related to the concept of delay discounting. Delay discounting refers to the fact that the value of a delayed reinforcer (i.e., or reward) is discounted compared to an immediate reinforcer. Principles of behavioral economics involving the process of delay discounting can provide an explanation for inconsistent choices, impulsivity and ambivalent feelings observed in addiction. Thus, as illustrated in Fig. 1B, ambivalence could be conceived as a consequence and clinical expression of delay discounting. It emerges from decision-making and delay-discounting processes in an environment where drugs exert a dual influence on individuals; short-term positive effects and long-term negative consequences. Ambivalence is however more complex than delay discounting and likely involves additional decision-making processes (for instance, decision-making under uncertainty).

**Fig. 2:**
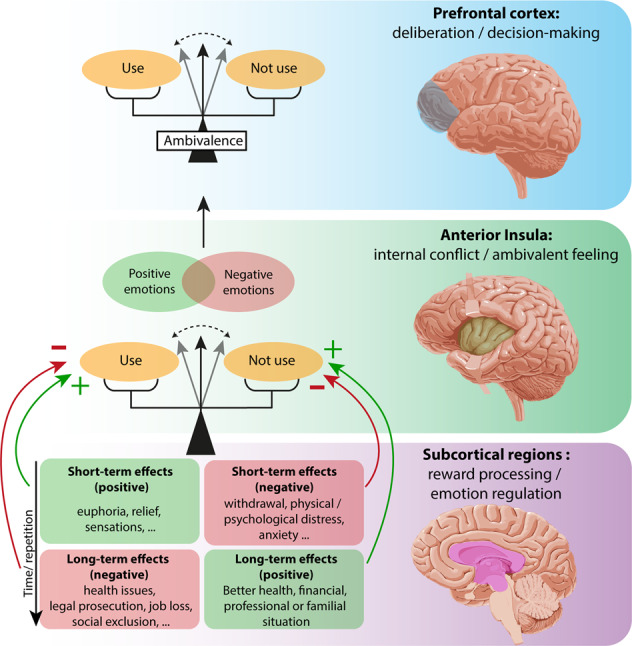
Neurobiology of ambivalence. Reward processing and emotion regulation involve subcortical regions, which include the ventral tegmental area, the striatum and the amygdala (bottom level). These regions contribute to assign subjective value and emotion to each option (using or not using). Notably, emotions of positive and negative valence are encoded in the amygdala [69, 70] and integrated in the anterior insula, which contributes to the subjective experience of internal conflict and ambivalent feeling (middle level). Information from subcortical regions and the insular cortex are integrated by cortical regions and notably the prefrontal cortex (higher level) to allow for option assessment, deliberation and appropriate decision-making.

Subcortical regions including the mesolimbic circuit, the striatum and the amygdala, are involved in reward processing and emotion regulation. These regions will contribute to associate subjective value and emotion to each option (using or not using). Notably, emotions of positive and negative valence are encoded in the amygdala [68, 69] and integrated in the anterior insula, which will contribute to the subjective experience of internal conflict and ambivalent feeling [70]. Finally, information from subcortical regions and the insula are integrated by the prefrontal cortex, implicated in weighing the options to compute their relative value and carry out a decision (Fig. 2) [71–74].

Importantly, although brain alterations induced by repeated drug exposure are likely involved in the development and maintenance of addiction, they are not required to explain ambivalence in the choice model presented here. Instead, it seems that short-term positive effects of drug and consideration of long-term negative consequences, combined with the anticipation of long-term benefits of abstinence, are responsible for ambivalent feelings.

### Jean Bernard, would it be possible to target this process and highlight the negative consequences of drug use while emphasizing the positive long-term benefits of abstinence in a therapeutic approach?

JBD: Yes, indeed. This is one of the principles of Motivational Interviewing (MI) (Box 3), which represents another step in the development of therapeutic strategies to address addictions, by focusing on the notion of motivation to change [75]. The concept underlying MI is that ambivalence about change—concurrently wanting to make a change while also feeling reticent to do so—is normal and central in addictions. An important consequence of the presence of ambivalence is that clinicians should avoid confronting and voicing arguments in favor of change directly, because this would result in patients showing reticence or voicing arguments for continuing drug or alcohol use, thus reinforcing their current behavior. Recognizing ambivalence allows the clinician to explore it, bringing patients to evoke arguments both favoring change (change talk) and opposing change (sustain talk). The strategic emphasis in MI is on purposefully evoking and reinforcing change talk (i.e., reasons, desire, ability and commitment to change), but also responding to sustain talk in a way that respects it, but does not strengthen or encourage it.

The therapeutic hypothesis of motivational interviewing is that patients need to hear themselves voicing the advantages of change and to encounter their own behavior and personal values in order to develop enough motivation to change. The therapists avoid confrontation and do not force abstinence (confrontational model), nor do they prematurely teach behavioral strategies to avoid craving and relapse (cognitive and behavioral treatment model). Instead, they act as a guide, eliciting patient aspirations and strengths, listening to them in the spirit of acceptance and non-judgment, and supporting their autonomy in decision-making. Research indicates that MI technical skills are associated with a higher proportion of change talk, which is predictive of actual change in behavior [76].

It is worth noting that the principles of MI imply that the patients have some ability and therefore some responsibility to change. When the moral perspective on addictions was predominant, the AA/NA philosophy and the brain disease model issued from neurosciences developed a symmetrical counterargument, pointing out the limitation or absence of responsibility to change in individuals suffering from addictions. Currently, a more balanced perspective on the treatment of addiction is possible, in which some degree of autonomy coexists with the ability and responsibility to change. This brings a positive therapeutic perspective, but also has important moral implications. Responsibility to change carries the risk that the person feels guilty for not changing, thus complicating the interactions with significant others and health care providers. In turn, those trying to help someone suffering from addiction, because of this responsibility and ability, might adopt MI non-adherent and counterproductive attitudes such as pressure, threats or constraints. MI therefore requires not only empathy, acceptance and autonomy support from the provider, but also, simultaneous acceptance that the person is responsible for deciding to change, or not.

To conclude our discussion, I think that a better understanding of the physiological processes explaining ambivalence, and, more generally, the mechanisms underlying addiction, is essential for clinicians in allowing them to provide “gentle” psychoeducation to patients. It also permits patients to better understand their ability to resist the strong pathophysiological processes underlying addiction.

### Youna, what did you get from this exchange?

YV: Our dialog reveals that while neurobiological theories of addiction informed us on the mechanisms responsible for the development of addiction, the medicine of addiction can enlighten preclinical research on the reality of drug use in addicted individuals and the interventions most effective at promoting remission. Therefore, this exchange allowed me to more fully comprehend the reciprocal influences and mutual enrichment between the preclinical research and addiction medicine. Furthermore, through this dialog, I learned about the motivational interviewing approach, which has aroused my interest in the cognitive and neurobiological bases of ambivalence, a topic largely overlooked in preclinical research on addiction. Perhaps this could be explained by the fact that non-human animals cannot report their feelings, as can humans. However, in my opinion, the progress already made in our understanding of decision-making and delay-discounting processes using animal models can be extended to the investigation of ambivalence. We can infer from their behavior whether rodents deliberate [77] or could recognize that choosing an alternative option would have been more valuable, akin to the human feeling of regret. Thus, I am sure that future research can find a proxy for ambivalence. Extending preclinical research to other species more evolved cognitively (i.e., non-human primates) could constitute a valuable resource toward this end. It was recently suggested that the translational validity of animal models could be improved with a reverse translational approach (Box 1) consisting of mimicking successful treatment in animals, in order to study the development and recovery from addiction in ecologically relevant settings [2]. In that respect, investigating the neurobiology of ambivalence could represent a new promising avenue for future research.

## Conclusion

Two years ago, Field and Kersbergen published an opinion article questioning the relevance of animal models of addiction [1]. The authors suggest that preclinical research has “not served us well in understanding and treating addiction in humans”, notably because of the poor translational predictive validity (Box 1) of animal models and the prevailing conceptualization of addiction (emerging from preclinical research) as a disorder of habit and compulsion. Although we agree to some extent with the first point, we strongly disagree with the second, and our opinion is supported by the dialog presented herein.

Regarding the poor predictive validity of animal models, it is worth pointing out that preclinical models have contributed to identifying several pharmacotherapies such as varenicline and buprenorphine [78, 79] (Box 3). This being said, decades of preclinical research have not contributed to significant advances in the development of new pharmacotherapies. This translational crisis could partly result from the use of preclinical experimental designs and endpoints that do not correctly model the reality of addiction in patients [2]. Furthermore, as we progress in our understanding of addiction, we measure the complexity and multi-faceted nature of this disorder, involving multiple paths, trajectories and highly specific neurobiological processes. Thus, trying to “repair” the brain is illusive. Pharmacological treatments can at best, temper craving and withdrawal, the most efficient approaches being through substitution (i.e., with nicotine or opiates) (Box 3). In our opinion, appropriate therapeutic approach should not aim at directly restoring addiction-induced brain alterations, but could indirectly do so, through psychotherapies (such as cognitive and behavioral therapies or motivational interviewing), which drive changes in behavior, learning and memory. Thus, although we believe that preclinical research on the development and improvement of pharmacotherapies is important, preclinical research should focus effort on more fundamental research on the cognitive and neurobiological mechanisms underlying addiction. From a clinical perspective, better understanding the cognitive and neurobiological bases of ambivalence could allow patients and health care providers better apprehending the condition of addiction and contribute to improve implementation of psychotherapies and reduce stigma. Further, fundamental knowledge on the cognitive mechanisms at play in addiction and targeted in psychotherapies could help clinicians to provide gentle psychoeducation to their patients in order to foster their ability to resist addiction pathophysiological processes.

With respect to the popular preclinical conceptualization of addiction as a disorder of compulsion arising from the brain disease model, we do not consider this as a limit of preclinical research. While the compulsive account of addiction appeared to justify that individuals had no responsibility for the occurrence and maintenance of their addictions, this belief was already in place in addiction medicine, when the brain disease model was first introduced in 1997 [3]. Ironically, preclinical research studies also contributed to the conceptualization of addiction as a disorder of choice and often bolster the arguments used by the detractors of the brain disease model to support their claim [47, 55, 80, 81]. Thus, as for the question “are animal models of addiction useful?”, our answer is yes. As previously reported [82], animal models are useful in understanding and explaining the neurobiological and cognitive processes involved in the development and recovery from addictions. Perhaps preclinical research does not contribute to the development of new treatments, but it does play a role in shaping theoretical conceptualizations of addiction and has considerable implications at individual, philosophical and societal levels. It can help improve clinical care, guide the implementation of therapeutic approaches, and strengthen the enactment of drug policies that optimize the desired beneficial outcomes.

Moving forward, we believe that more discussion and collaboration between preclinical researchers and clinicians is required to take into account the complexity of drug use in humans suffering from addiction when modeling this disorder, or at least some of its key symptoms, in non-human animals. On the other hand, preclinical research on the cognitive and neurobiological mechanisms underlying addiction can guide psychotherapies and help clinicians in understanding the nature of patients’ internal conflicts, which can in turn be explained to the patients. Through this dialog, we realized that although we share the same concepts and terminology, clinicians and preclinical researchers do not assign the same meaning to these concepts (i.e., compulsion). Thus, to improve translational utility of animal model and treatments, the future direction we suggest is to promote exchange between the two disciplines through (1) training (i.e., include clinical courses in neuroscience degree, and neuroscience courses in medical degree) (2) international conferences on translational psychiatry (involving both clinicians and preclinical researchers) and (3) funding of translational research with collaboration between preclinical and clinical researchers. The disconnection between preclinical research and addiction medicine could partly result from the “rodent-centric” preclinical view of addiction, at the foundation of the BDMA. Thus, future training and research directions should absolutely include preclinical and clinical studies from numerous species, including non-human primates.
